# Reduced fibrin clot permeability on admission and elevated E-selectin at 3 months as novel risk factors of residual pulmonary vascular obstruction in patients with acute pulmonary embolism

**DOI:** 10.1007/s11239-023-02901-y

**Published:** 2023-11-06

**Authors:** Konrad Stępień, Michał Ząbczyk, Magdalena Kopytek, Joanna Natorska, Jarosław Zalewski, Anetta Undas

**Affiliations:** 1 Department of Thromboembolic Disorders, Institute of Cardiology, Jagiellonian University Medical College, Kraków, Poland; 2 Department of Coronary Artery Disease and Heart Failure, John Paul II Hospital, Kraków, Poland; 3 Krakow Centre for Medical Research and Technologies, John Paul II Hospital, Kraków, Poland; 4 Department of Coronary Artery Disease and Heart Failure, Institute of Cardiology, Jagiellonian University Medical College, Kraków, Poland

**Keywords:** Residual pulmonary vascular obstruction, Pulmonary embolism, Fibrin clot, Thrombin generation, E-selectin

## Abstract

**Background:**

Residual pulmonary vascular obstruction (RPVO) is common following pulmonary embolism (PE) but its association with fibrin clot properties is poorly understood. We investigated whether prothrombotic state and hypofibrinolysis markers can identify patients with RPVO.

**Methods:**

In 79 normotensive noncancer patients (aged 56 ± 13.3 years) with acute PE, we determined fibrin clot permeability (K_s_), clot lysis time (CLT), endogenous thrombin potential (ETP), fibrinolysis proteins, oxidative stress markers, and E-selectin on admission before initiation of anticoagulant therapy, after 5–7 days, and 3 months of anticoagulation. RPVO was diagnosed using computed tomography angiography 3–6 months since PE.

**Results:**

Patients with RPVO (n = 23, 29.1%) had at baseline higher simplified Pulmonary Embolism Severity Index (sPESI) (P = 0.004), higher N-terminal brain natriuretic propeptide (P = 0.006) and higher D-dimer (P = 0.044). Patients with versus without RPVO had lower K_s_ (P < 0.001) and longer CLT (P < 0.05), both at baseline and 5–7 days since admission, but not at 3 months. Patients with RPVO showed 40.6% higher E-selectin (P < 0.001) solely at 3 months. By multivariable logistic regression, baseline K_s_ (odds ratio [OR] 0.010, 95% confidence interval [CI] 0.001–0.837, P = 0.042, per 10^− 9^ cm^2^), baseline D-dimer (OR 1.105, 95% CI 1.000-1.221, P = 0.049, per 100 ng/ml), and E-selectin levels after 3 months (OR 3.874, 95% CI 1.239–12.116, P = 0.020, per 1 ng/ml) were associated with RPVO.

**Conclusions:**

RPVO patients despite anticoagulation characterize with the formation of denser fibrin clots on admission and higher E-selectin at 3 months. Those parameters could be the potential novel RPVO risk factors that warrant further evaluation in an independent cohort.

**Graphical Abstract:**

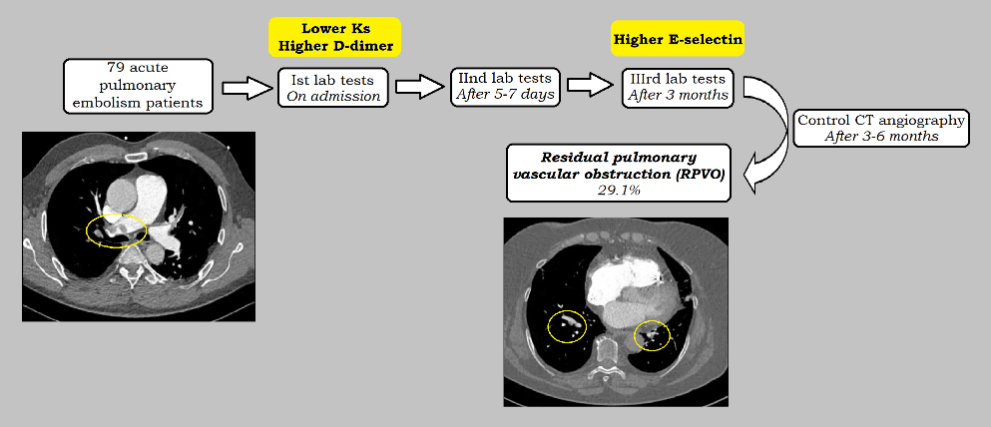

**Supplementary Information:**

The online version contains supplementary material available at 10.1007/s11239-023-02901-y.

## Key points

- RPVO occurs in a large proportion of acute PE patients despite anticoagulation.
- Plasma fibrin clot permeability and lysability on admission and after 5–7 days are potential novel RPVO risk factors.
- Elevated E-selectin after 3 months since PE was observed in RPVO patients.
- E-selectin inhibitors are potentially attractive in the RPVO prevention in PE patients.

**Supplementary Information:**

The online version contains supplementary material available at 10.1007/s11239-023-02901-y.

## Introduction

Residual pulmonary vascular obstruction (RPVO) is a long-term complication of pulmonary embolism (PE) and is defined as residual perfusion defects after a currently recommended course of anticoagulant treatment. Its incidence ranges from 15 to 30% of PE patients [1]. As has been recently shown RPVO, along with unprovoked PE, is an independent risk factor of recurrent venous thromboembolism (VTE) [2]. Moreover, RPVO is one of the main determinants of the clinically significant post-PE syndrome [3]. Based on the literature the predictors of the occurrence of RPVO are: higher baseline obstruction level of pulmonary arteries, age ≥ 65 years, unprovoked PE, and chronic respiratory failure [4].

Growing evidence indicates that both deep vein thrombosis (DVT) and PE are associated with altered fibrin clot properties including impaired fibrinolytic capacity [5–7]. However, it is unclear whether prothrombotic clot properties contribute to RPVO. We have previously reported unfavorably altered fibrin clot properties in DVT patients with incomplete vein recanalization [8], reflected by 14.1% lower clot permeability and 11.3% longer lysis time. Moreover, we have recently suggested that oxidative stress and prothrombotic fibrin clot properties could be involved in the pathogenesis of the post-PE syndrome [9]. Lami et al. have shown that PE patients after one year or at least one month after anticoagulation withdrawal with RPVO > 10% in control lung scintigraphy had significantly longer lysis time and higher levels of plasminogen activator inhibitor-1 (PAI-1) than those with perfusion defects < 10% [1]. In turn, Planquette et al. reported that fibrinogen Bβ-chain monosialylation is useful for prediction of RPVO occurrence and they hypothesized that fibrin structure may contribute to the risk of developing RPVO [10].

E-selectin is a glycoprotein that facilitates thrombosis, directly modulating neutrophil and monocyte activity [11]. E-selectin knockout mice had decreased fibrin content of the thrombus and less vein wall inflammation [12]. Moreover, E-selectin is expressed later than P-selectin in the endothelium, approximately 2 days after the DVT occurrence [13]. It has been reported that plasma E-selectin levels in acute PE are reduced [14] or only slightly elevated [15] likely due to its breakdown in lysosomes shortly after translation [16]. Elevated E-selectin levels have been shown in post-thrombotic syndrome [17]. To our knowledge, there have been no studies linking E-selectin with RPVO.

Given a rather poor performance of available predictors of RPVO, we sought to investigate several prothrombotic and hypofibrinolytic markers, along with selectins as potential risk factors of RPVO in PE patients excluding those with high-risk PE.

## Materials and methods

In the current study we assessed 79 non-cancer and hemodynamically stable PE patients recruited from December 2016 to March 2021 and described in detail previously [9, 18]. PE was diagnosed based on the occurrence of typical clinical symptoms confirmed by computed tomography angiography (angio-CT). The simplified PE severity index (sPESI) was assessed initially in all patients [19]. Invasive evaluation of pulmonary pressure was not performed in any subject due to the low probability of chronic thromboembolic pulmonary hypertension, (CTEPH) on transthoracic echocardiography (TTE) [20]. DVT was diagnosed within the first 48 h since enrolment based on a positive finding of color duplex sonography. Provoked VTE was diagnosed if a patient had surgery requiring general anesthesia, major trauma, plaster cast or hospitalization in the past month, pregnancy or delivery in the past 3 months. RV dysfunction and comorbidities were defined as described previously [18].

RPVO was defined as residual perfusion defects on control computed tomography angiography performed after 3–6 months of anticoagulation [1]. Post-PE syndrome, diagnosed at 6 months since the index PE event, was defined by persistent dyspnea reported at 3 and 6 months since the event (New York Heart Association [NYHA] class II or more) and impaired exercise capacity using the respective reference values [9]. The Jagiellonian University Medical College Ethical Committee approved the study, and participants provided written informed consent in accordance with the Declaration of Helsinki.

### Laboratory investigations

All subjects were evaluated on admission before initiation of anticoagulant therapy and after 5–7 days. Blood samples were drawn from an antecubital vein with minimal stasis. Blood cell count, glucose, fibrinogen, high-sensitivity C-reactive protein (hsCRP), lipid profile, D-dimer, and factor (F)VIII activity were assayed by routine laboratory techniques in the hospital laboratory. N-terminal B-type natriuretic propeptide (NT-proBNP), high-sensitivity troponin T (TnT) were assessed by routine laboratory techniques in the hospital laboratory, while E-selectin, interleukin-6 (IL-6), L-lactate and 8-isoprostane were assayed by the immunoenzymatic tests (ELISA; R&D Systems, Abingdon, United Kingdom; Quantikine, R&D Systems, Minneapolis, USA; Abcam, Cambridge, United Kingdom; Cayman Chemical, Ann Arbor, MI, USA). Positive TnT was defined as a value > 14 pg/mL [21].

At 3 months of anticoagulant therapy blood samples were drawn 24–28 h since the administration of the last dose of direct oral anticoagulants (DOACs) and samples were evaluated if the drug concentration was below 30 ng/ml [22]. A chromogenic assay was used to measure anti-factor X (FXa) activity (BIOPHEN, Hyphen-Biomed, Neuville-Sur-Oise, France) in patients who received rivaroxaban or apixaban. In patients treated with warfarin, blood samples were drawn 24 h after the last dose of low-molecular-weight heparin. To evaluate efficiency of fibrinolysis, PAI-1 antigen, thrombin activatable fibrinolysis inhibitor (TAFI) activity (both from Hyphen-Biomed, Neuville-Sur-Oise, France), α2-antiplasmin, and plasminogen activity were measured (both Berichrom, Siemens Healthcare Diagnostics, Marburg, Germany).

The endogenous thrombin potential (ETP) was measured using calibrated automated thrombography (Thrombinoscope BV, Maastricht, the Netherlands). For fibrin clot analysis, blood samples (vol/vol, 9:1 of 3.2% trisodium citrate) were spun at 2500 g for 20 min and the supernatant was aliquoted and stored at -80 °C. All measurements were performed by technicians blinded to the origin of the samples. Intra-assay and inter-assay coefficients of variation were 5–7%. Fibrin clot permeation (Ks), reflecting the average pore size in the fibrin network was determined using a pressure-driven system as described previously [23]. Briefly, 20 mM CaCl_2_ and 1 U/mL human thrombin (Merck, Darmstadt, Germany ) were added to

citrated plasma. Volume of the buffer flowing through the clots was measured within 60 min. Fibrinolysis capacity (clot lysis time, CLT) was measured according to Pieters et al. [24]. Briefly, citrated plasma was mixed with 15 mM calcium chloride, human thrombin (Merck) at a final concentration of 0.5 U/ml, 10 µM phospholipid vesicles, and 18 ng/ml recombinant tPA (Boehringer Ingelheim, Ingelheim, Germany). A turbidity of the mixture was measured at 405 nm. Intra-assay and interassay coefficients of variation for the two fibrin variables were < 5% and < 8%, respectively.

### Statistical analysis

Variables were presented as numbers and percentages or median and interquartile range (IQR), as appropriate. Normality was assessed by Shapiro-Wilk test. Differences between the groups were compared using the Student’s t-test for normally distributed variables. In turn, the Mann-Whitney U-test was used for non-normally distributed variables. Categorical variables were compared by chi-squared test or Fisher’s exact test. Associations between parametric variables were assessed by the Pearson’s correlation test while between nonparametric by Spearman’s rank correlation coefficient. All independent variables potentially associated with both the exposure and outcome were included in the multivariable logistic regression to find parameters independently associated with RPVO. The best cut-off value that maximizes sensitivity and specificity of K_s_ and CLT for RPVO prediction was calculated by using the Receiver Operating Characteristics (ROC) curves. A two-sided P < 0.05 was considered statistically significant. All statistical analyses were performed using STATISTICA software Version 13.3 (StatSoft, Krakow, Poland) or IBM SPSS Statistics Version 26.0 (IBM Corp, Armonk, NY, USA).

## Results

Among 79 normotensive noncancer PE patients (aged 56 ± 13.3 years) 63.3% had unprovoked events (Table 1). Based on the angio-CT performed after 4 ± 1 months since the diagnosis of PE and after at least 3 months of oral anticoagulation therapy (rivaroxaban – 77.2%, apixaban – 19.0%, warfarin – 3.8%), we detected RPVO in 23 individuals (29.1%).

**Table 1:** Baseline characteristics of pulmonary embolism patients with residual pulmonary vascular obstruction (RPVO) compared to those without RPVO.

	RPVO (n = 23)	Non-RPVO (n = 56)	P-value
Age, y	54 (50–62)	58.5 (45.5–67.5)	0.41
Male, n (%)	13 (56.5)	34 (60.7)	0.73
Body-mass index, kg/m^2^	27 (24–28)	27 (24–30)	0.61
Early mortality risk, n (%)			
Low	1 (4.4)	17 (30.4)	
Intermediate-low	15 (65.2)	34 (60.7)	**0.007**
Intermediate-high	7 (30.4)	5 (8.9)	
sPESI			
0	1 (4.4)	17 (30.4)	
1	6 (26.1)	21 (37.5)	**0.004**
2	11 (47.8)	16 (28.6)	
3	5 (21.7)	2 (3.6)	
Embolic material distribution, n (%)			
Saddle	9 (39.1)	14 (25.0)	0.21
Bilateral	21 (91.3)	46 (82.1)	0.30
Central	18 (78.3)	36 (64.3)	0.23
Segmental	3 (13.0)	10 (17.9)	0.60
Subsegmental	2 (8.7)	10 (17.9)	0.30
Positive troponin T, n (%)	9 (39.1)	7 (12.5)	**0.007**
RV dysfunction, n (%)	11 (47.8)	12 (21.4)	**0.019**
First PE, n (%)	22 (95.7)	51 (91.1)	0.49
Provoked PE, n (%)	7 (30.4)	22 (39.3)	0.46
Comorbidities, n (%)			
Arterial hypertension	15 (65.2)	28 (50.0)	0.22
Diabetes mellitus	10 (43.5)	20 (35.7)	0.52
Chronic kidney disease	6 (26.1)	16 (28.6)	0.82
Coronary artery disease	9 (39.1)	29 (51.8)	0.31
Heart failure	7 (30.4)	15 (26.8)	0.74
Previous stroke	1 (4.4)	5 (8.9)	0.49
Concomitant medications, n (%)			
Aspirin	6 (26.1)	22 (39.3)	0.27
Statins	21 (91.3)	40 (71.4)	0.06
Anticoagulation			
Rivaroxaban	16 (69.6)	45 (80.4)	0.16
Apixaban	7 (30.4)	8 (14.3)	
Warfarin	0 (0.0)	3 (5.4)	
Basic laboratory investigations			
Hemoglobin, g/dL	13.7 (12.8–15.2)	13.9 (13.2–14.9)	0.89
White blood cells, 10^3^/µL	6.7 (6.1–7.5)	6.0 (5.0-7.2)	0.053
Platelets, 10^3^/µL	245 (207–314)	225 (189–280)	0.26
Glucose, mmol/L	5.7 (5.4–6.2)	5.8 (5.2–6.5)	0.99
NT-proBNP, pg/mL	634 (391–1261)	290 (105–796)	**0.006**
LDL cholesterol, mg/dL	80 (70–107)	87 (76–102)	0.43
High-sensitivity CRP, mg/L	2.8 (1.7–5.6)	1.9 (1.4–5.5)	0.24
Coagulation variables at baseline			
Fibrinogen, g/L	3.2 (2.6–4.1)	3.2 (2.6–3.7)	0.35
D-dimer, ng/mL	3372 (2015–5940)	2187 (1337–3981)	**0.044**
PAI-1, ng/mL	29.5 (19.5–42.4)	22.5 (17.2–30.8)	**0.045**
TAFI activity, %	105 (95–114)	102 (95–117)	0.83
α2-antiplasmin, %	98 (90–111)	108 (97–116)	0.21
Plasminogen, %	101 (93–114)	107 (99–117)	0.24
Factor VIII, %	142 (120–178)	142 (119–172)	0.91
ETP, nM×min	1724 (1510–1976)	1582 (1464–1721)	0.07
K_s_, ×10^− 9^cm^2^	5.9 (5.6–6.4)	7.3 (6.9–7.6)	**< 0.001**
CLT, min	111 (98–124)	99 (86–109)	**0.023**
Other parameters			
E-selectin, ng/mL	28.9 (26.2–38.7)	28.9 (25.9–32.0)	0.36
Interleukin-6, pg/mL	4.0 (3.4–4.2)	3.7 (3.3–4.1)	0.99
L-lactate, mM	2.3 (2.0-3.1)	2.0 (1.7–2.4)	**0.045**
8-isoPGF-2a, pg/mL	428 (345–553)	337.5 (296-517.5)	**0.046**

Categorical variables are presented as numbers (percentages). Continuous variables are expressed as median (interquartile range)

Abbreviations: RPVO, Residual pulmonary vascular obstruction; sPESI, Simplified Pulmonary Embolism Severity Index; RV, Right ventricle; PE, Pulmonary embolism; NT-proBNP, N-terminal brain natriuretic propeptide; LDL, Low-density lipoprotein; CRP, C-reactive protein; PAI-1, Plasminogen activator inhibitor type 1; TAFI, Thrombin activatable fibrinolysis inhibitor; ETP, Endogenous thrombin potential; K_s_, Plasma fibrin clot permeability; CLT, Plasma clot lysis time; isoPGF-2a, 8-iso-prostaglandin F2 alpha

### At baseline

As shown in Table 1, at baseline the RPVO group, as compared to the non-RPVO group, had a more severe PE manifestation as reflected by the early mortality risk assessment (P = 0.007) and sPESI (P = 0.004), along with increased prevalence of positive TnT (P = 0.007) and RV dysfunction (P = 0.019). There were no differences in the initial embolic material location. In both groups cardiovascular risk factors were similarly distributed (Table 1).

**Fig. 1:**
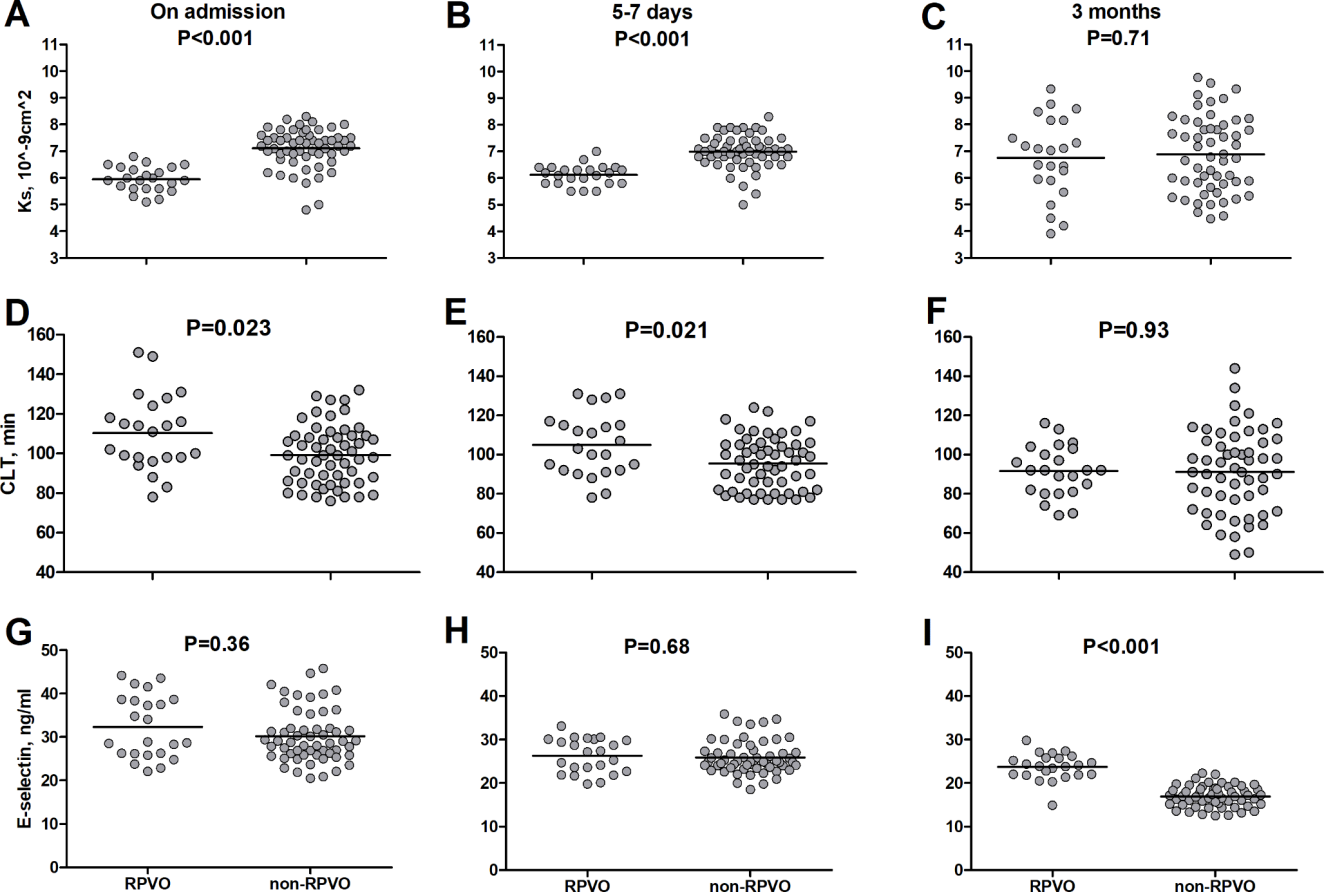
K_s_, CLT and E-selectin in pulmonary embolism patients with and without residual pulmonary vascular obstruction (RPVO) on admission and during follow-up. Abbreviations: RPVO, residual pulmonary vascular obstruction; K_s_, plasma fibrin clot permeability; CLT, plasma clot lysis time

Regarding laboratory investigations, higher D-dimer (by 54%, P = 0.044) and NT-proBNP (by 119%, P = 0.006) levels were noted in the RPVO compared to the non-RPVO group (Table 1). Despite similar fibrinogen concentrations and ETP, patients with RPVO had 19.2% lower K_s_ (P < 0.001), and CLT prolonged by 12% (P = 0.023) (Fig. 1A and D) in association with 31.1% higher PAI-1 antigen (P = 0.045) compared to those without RPVO (Table 1). Moreover, the L-lactate levels were 15% higher (P = 0.045) and 8-isoprostane levels by 21.1% (P = 0.046) in the RPVO group. 8-isoprostane showed positive correlation with hsCRP at baseline (R = 0.45, P = 0.001). There were no differences in other fibrinolysis proteins and oxidative stress markers assessed at baseline (Table 1).

### 5–7 days since admission

After 5–7 days in RPVO patients lower K_s_ (6.2 [5.8–6.4] vs. 7.0 [6.8–7.4] ×10^− 9^cm^2^, P < 0.001) and prolonged CLT (103 [92–115] vs. 96 [82–105] min, P = 0.021) were observed and both fibrin measures were inversely correlated (R=-0.32, P = 0.004) (Table 2; Fig. 1B and E). Among other routine laboratory variables tested at this time point, only D-dimer levels were higher by 37% (P = 0.044) in RPVO patients. Like on admission, at this time point K_s_ was lower by 14% (P < 0.001) and CLT was longer by 11% (P = 0.017) in patients with RPVO versus without RPVO.

**Table 2:** Laboratory parameters during follow-up

	RPVO (n = 23)	Non-RPVO (n = 56)	P-value
5–7 days			
Fibrinogen, g/L	2.9 (2.5–3.9)	3.0 (2.5–3.3)	0.33
K_s_, ×10^− 9^cm^2^	6.2 (5.8–6.4)	7 (6.8–7.4)	**< 0.001**
CLT, min	103 (92–115)	96 (82–105)	**0.021**
E-selectin, ng/mL	27.2 (22.7–30.1)	24.9 (23.6–27.4)	0.68
8-isoPGF-2a, pg/mL	268 (231–292)	226 (195–281)	0.24
3 months			
Fibrinogen, g/L	2.8 (2.5–3.1)	2.8 (2.5–3.2)	0.73
D-dimer, ng/mL	264 (205–291)	262 (220–344)	0.37
PAI-1, ng/mL	20.0 (17.0-30.1)	20.0 (15.2–25.3)	0.25
ETP, nM×min	1213 (1173–1318)	1263 (1053–1382)	0.85
K_s_, ×10^− 9^cm^2^	7.0 (5.9–8.2)	6.8 (5.8-8.0)	0.71
CLT, min	92 (81–103)	91 (72–107)	0.93
E-selectin, ng/mL	23.9 (21.9–25.8)	17.0 (15.2–18.6)	**< 0.001**
8-isoPGF-2a, pg/mL	100 (94–112)	104 (89–112)	0.88
High-sensitivity CRP, mg/L	1.4 (1.0-2.9)	1.3 (0.9–2.8)	0.56
Interleukin-6, pg/mL	3.6 (2.3–4.6)	2.5 (2.1-4.0)	0.21

Continuous variables are expressed as median (interquartile range)

Abbreviations: RPVO, residual pulmonary vascular obstruction; K_s_, plasma fibrin clot permeability; CLT, plasma clot lysis time; 8-isoPGF-2a, 8-iso-prostaglandin F2 alpha; PAI-1, plasminogen activator inhibitor type 1; ETP, endogenous thrombin potential; CRP, C-reactive protein

### At 3 months

Comparison of the RPVO vs. non-RPVO patients after 3 months of anticoagulation showed solely a few intergroup differences in laboratory parameters. The differences in K_s_ and CLT were no longer observed after 3 months (Table 2; Fig. 1C F). However, after 3 months, despite the lack of differences at baseline and after 5–7 days, 40.6% higher values of E-selectin were observed in the RPVO group (Table 2; Fig. 1G H and 1I). Of note, E-selectin at 3 months was inversely correlated with baseline K_s_ (R=-0.57, P < 0.001) and K_s_ after 5–7 days (R=-0.52, P < 0.001), but not with K_s_ measured at 3 months since PE. Similarly, E-selectin showed positive correlations with CLT at baseline (R = 0.31, P = 0.006) and after 5–7 days (R = 0.30, P = 0.008), but not at 3 months. E-selectin was positively associated solely with IL-6, but not with hsCRP or fibrinogen, determined at 3 months (R = 0.34, P = 0.002). There were no differences in other laboratory parameters measured at 3 months between RPVO and non-RPVO patients (Table 2).

### RPVO and post-PE syndrome

The post-PE syndrome was observed in 26 patients (32.9%). After 6 months there was no difference in the incidence of post-PE syndrome between the RPVO and non-RPVO groups (9 [39.1%] vs. 17 [30.9%], P = 0.48). In general, RPVO and post-PE syndrome occurrence were associated with increased baseline clinical risk (Table 3). The patients with both RPVO and post-PE syndrome were characterized by higher incidence of positive TnT (P = 0.018) and RV dysfunction (P < 0.001) (Table 3). The coexistence of RPVO and post-PE syndrome was associated with increased baseline NT-proBNP, E-selectin, PAI-1, ETP, CLT and reduced K_s_ values (Table 3). RPVO combined with post-PE syndrome was associated with lower K_s_ (P < 0.001) and prolonged CLT (P = 0.004) after 5–7 days as well as with increased E-selectin (P < 0.001) levels and longer CLT (P = 0.001) after 3 months (Table 3).

**Table 3:** Coexistence of residual pulmonary vascular obstruction and post-pulmonary embolism syndrome: subgroup analysis.

	RPVO(+) post-PE(+) (n = 9)	RPVO(+) post-PE(-) (n = 14)	RPVO(-) post-PE(+) (n = 17)	RPVO(-) post-PE(-) (n = 39)	P-value
Early mortality risk, n (%)					
Low	0 (0.0)^*^	1 (7.1)^*^	2 (11.8)^*^	15 (38.5)	
Intermediate-low	4 (44.4)	11 (78.6)	12 (70.6)	22 (56.4)	**0.001**
Intermediate-high	5 (55.6)^#, ^, *^	2 (14.3)	3 (17.7)	2 (5.1)	
sPESI					
0	0 (0.0)^*^	1 (7.1)	2 (11.8)	15 (38.5)	
1	1 (11.1)	5 (35.7)	5 (29.4)	16 (41.0)	**< 0.001**
2	4 (44.4)	7 (50.0)^*^	9 (52.9)^*^	7 (18.0)	
3	4 (44.4)^#, ^, *^	1 (7.1)	1 (5.9)	1 (2.6)	
Positive troponin T, n (%)	5 (55.6)^^, *^	4 (28.6)	3 (17.7)	4 (10.3)	**0.018**
RV dysfunction, n (%)	8 (88.9)^#, ^, *^	3 (21.4)	8 (47.1)	4 (10.3)	**< 0.001**
NT-proBNP, pg/mL	987 (450–1261)^*^	507 (391–996)	501 (229–1241)	232 (92–705)	**0.013**
E-selectin, ng/mL	38.4 (37.3–41.6)^#, *^	27.3 (25.8–28.9)	31.5 (26.9–39.7)^*^	28.0 (25.8–31.2)	**0.003**
PAI-1, ng/mL	30.0 (29.7–45.8)^*^	27.7 (16.7–32.8)	28.0 (19.9–36.0)^*^	22.2 (14.1–29.7)	**0.014**
ETP, nM×min	1788 (1663–1976)^*^	1663 (1437–1765)	1683 (1513–1870)^*^	1502 (1436–1657)	**0.015**
K_s_, ×10^− 9^cm^2^	6.3 (5.8–6.5)^^, *^	5.9 (5.6-6.0)^^, *^	7.3 (6.8–7.5)	7.2 (6.9–7.6)	**< 0.001**
CLT, min	116 (114–130)^#, ^, *^	99 (94–115)	104 (94–113)	97 (85–108)	**0.005**
5–7 days					
E-selectin, ng/mL	28.7 (27.2–30.1)	24.2 (21.9–29.4)	25.9 (24.3–30.0)	24.8 (23.4–26.8)	0.09
K_s_, ×10^− 9^cm^2^	6.3 (6.2–6.4)^^, *^	6.1 (5.8–6.3)^^, *^	7.0 (6.8–7.1)	7.1 (6.5–7.5)	**< 0.001**
CLT, min	112 (107–128)^#, ^, *^	95 (90–114)	101 (90–108)^*^	94 (80–103)	**0.004**
3 months					
E-selectin, ng/mL	24.1 (22.8–25.8)^^, *^	23.6 (21.4–25.7)^^, *^	18.1 (15.9–19.2)	16.7 (14.7–18.3)	**< 0.001**
ETP, nM×min	1296 (1174–1318)	1198 (1173–1292)	1302 (1085–1402)	1245 (1053–1318)	0.69
K_s_, ×10^− 9^cm^2^	7.2 (6.4–8.2)	6.5 (5.0-7.3)	6.1 (5.2–7.5)	7.3 (5.9–8.2)	0.15
CLT, min	103 (89–105)	91 (81–96)^^^	106 (98–114)^*^	84 (69–98)	**0.001**

Categorical variables are presented as numbers (percentages). Continuous variables are expressed as median (interquartile range). ^#^ P < 0.05 vs. RPVO(+) post-PE(-), ^^^ P < 0.05 vs. RPVO(-) post-PE(+), ^*^ P < 0.05 vs. RPVO(-) post-PE(-)

Abbreviations: RPVO, residual pulmonary vascular obstruction; post-PE, post-pulmonary embolism syndrome; sPESI, simplified Pulmonary Embolism Severity Index; RV, right ventricle; NT-proBNP, N-terminal brain natriuretic propeptide; PAI-1, plasminogen activator inhibitor type 1; ETP, endogenous thrombin potential; K_s_, plasma fibrin clot permeability; CLT, plasma clot lysis time

### Multivariable analysis

Before RPVO inclusion into the multivariate model as a dependent variable, all significant associations between independent covariates were identified. Patients with RV dilatation more frequently were TnT positive (P < 0.001) and the mean plasma NT-proBNP was higher in patients with sPESI ≥ 1 versus 0 (P < 0.001). Moreover, E-selectin levels were associated with sPESI (P < 0.001), RV dilatation (P = 0.001) and positive TnT (P = 0.001). By multivariable logistic regression (Table 4), baseline K_s_ (P = 0.042), baseline D-dimer (P = 0.049), and E-selectin levels after 3 months (P = 0.020) influenced the presence of RPVO (R^2^ Nagelkerke 0.92).

**Table 4:** Multivariable logistic regression with residual pulmonary vascular obstruction as a dependent variable (Nagelkerke R2 0.920, Hosmer Lemenshow P = 0.99)

	Univariable analysis					Multivariable analysis			
	OR	95% CI for OR		P-value	OR		95% CI for OR		P-value
Age, per 1 y	0.992	0.957	1.029	0.662	0.884		0.715	1.093	0.255
RV dilatation, yes vs. no	3.361	1.191	9.487	**0.022**	2.30		0.071	73.37	0.636
Positive troponin T, yes vs. no	4.500	1.421	14.248	**0.011**					
sPESI 0 vs. ≥ 1	9.590	1.194	77.022	**0.033**	105.5		0.241	4592.5	0.104
NT-proBNP, per 1 pg/mL	1.001	1.000	1.001	**0.034**					
K_s_, per 10^− 9^ cm^2^	0.093	0.032	0.269	**< 0.001**	0.010		0.001	0.837	**0.042**
D-dimer, per 100 ng/mL	1.019	0.999	1.040	0.066	1.105		1.000	1.221	**0.049**
E-selectin 3 months, per 1 ng/mL	2.535	1.616	3.977	**< 0.001**	3.874		1.239	12.116	**0.020**

Abbreviations: OR, odds ratio; CI, confidence interval; RV, right ventricle; sPESI, simplified Pulmonary Embolism Severity Index; NT-proBNP, N-terminal brain natriuretic propeptide; Ks, plasma fibrin clot permeability

### New biomarkers in prediction of RPVO

**Fig. 2:**
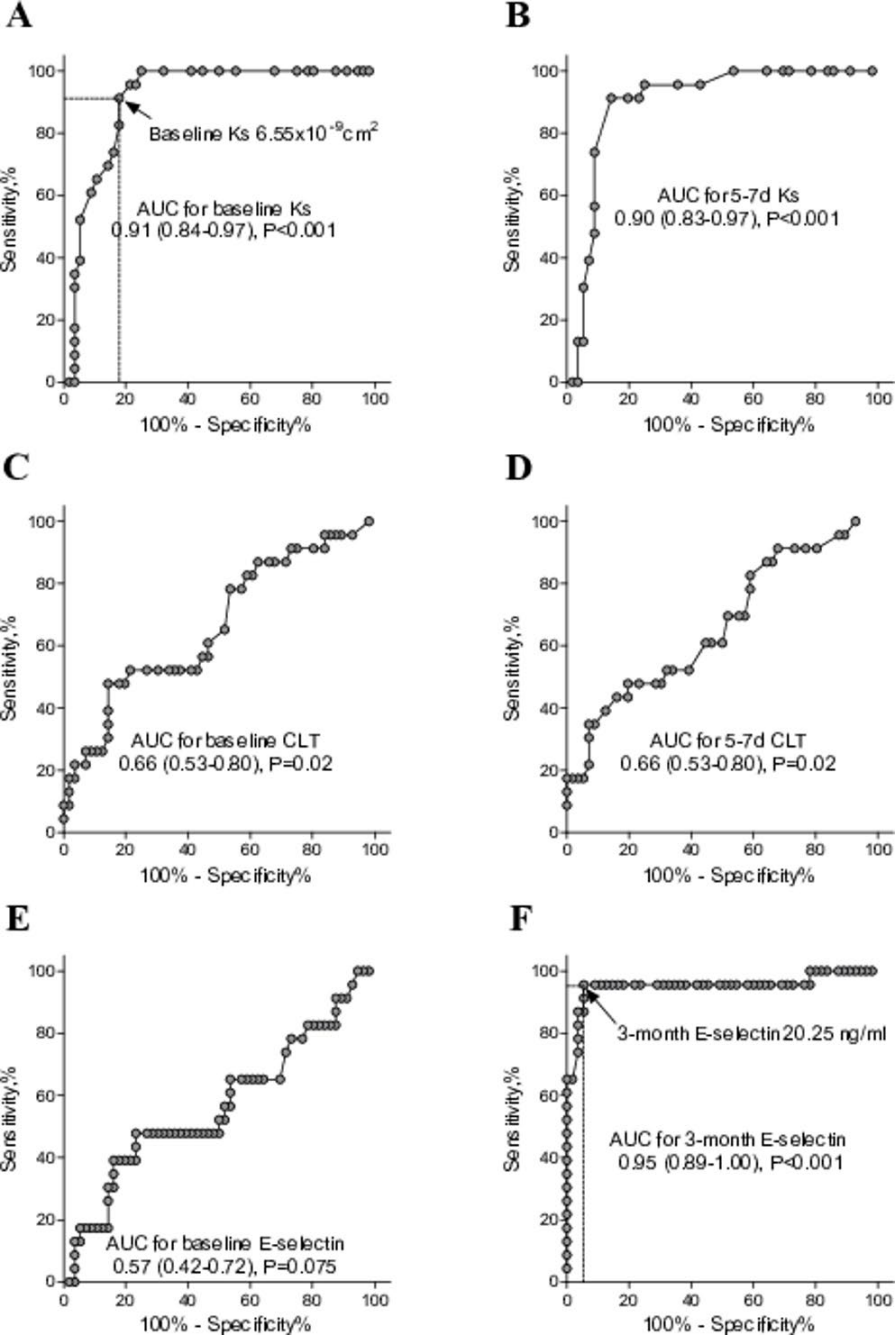
The receiver operating characteristics curves of RPVO occurrence for baseline K_s_**(A)**, K_s_ after 5–7 days **(B)**, baseline CLT **(C)**, CLT after 5–7 days **(D)**, baseline E-selectin **(E)** and 3-month E-selectin **(F)** with cut-off values for baseline K_s_ and 3-month E-selectin. Abbreviations: K_s_, plasma fibrin clot permeability; CLT, plasma clot lysis time; AUC, area under the curve

Baseline K_s_ reached the area under the ROC curve (AUC) of 0.91 (95% CI 0.84–0.97) for prediction of RPVO presence with a cut-off value of < 6.55 × 10^− 9^ cm^2^ and a sensitivity of 91.3% and specificity of 83.9% (Fig. 2A, Supplementary Table 1). As few as 2 of 23 patients with RPVO had baseline K_s_ higher than this value, and 9 of 56 patients without RPVO had lower baseline K_s_ than the cut-off (Supplementary Table 1). In turn, K_s_ as measured 5–7 days after PE diagnosis reached the AUC of 0.90 (95% CI 0.83–0.97) for prediction of RPVO presence with a cut-off value of < 6.45 × 10^− 9^cm^2^ and a sensitivity of 91.0% and specificity of 86.0% (Fig. 2B). Similarly, both baseline CLT and CLT after 5–7 days reached only the AUC of 0.66 (95% CI 0.53–0.80) (Fig. 2C and D). E-selectin measured 3 months following PE reached the AUC of 0.95 (95% CI 0.89-1.00) for prediction of RPVO presence with a cut-off value > 20.25 ng/ml and a sensitivity of 95.6% and specificity of 94.6% (Fig. 2E F, Supplementary Table 1). A single individual of 23 patients with RPVO had 3-month E-selectin lower than this value and 3 of 56 patients without RPVO had 3-month E-selectin higher than this cut-off (Supplementary Table 1).

**Fig. 3:**
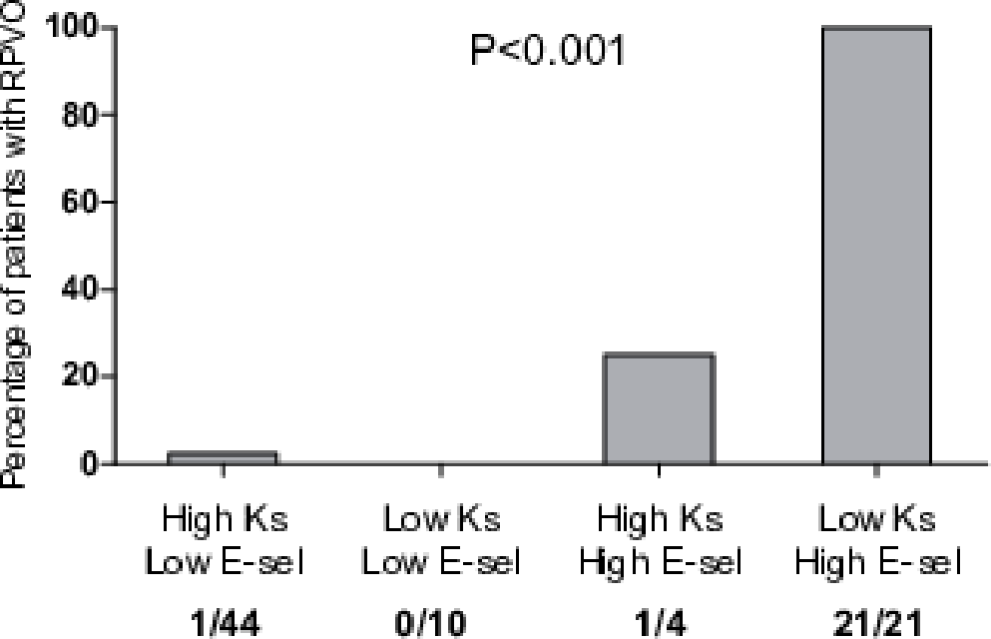
Simultaneous assessment of baseline K_s_ and 3-month E-selectin for prediction of RPVO. Abbreviations: K_s_, plasma fibrin clot permeability; E-sel, 3-month E-selectin; low K_s_, < 6.55 × 10^− 9^cm^2^; high K_s_, ≥ 6.55 × 10^− 9^cm^2^; low E-sel, ≤ 20.25 ng/ml; high E-sel, > 20.25 ng/ml. P-value for differences among four groups in chi-squared test; n/N, number of patients with RPVO to all patients in each subgroup

Based on cut-off values of baseline K_s_ and 3-month E-selectin, four groups have been created. RPVO was observed in all patients with low baseline K_s_ of less than 6.55 × 10^− 9^cm^2^ and with high 3-month E-selectin of more than 20.25 ng/ml, while only in 1 patient with high baseline K_s_ ≥ 6.55 × 10^− 9^ cm^2^ and low 3-month E-selectin ≤ 20.25 ng/ml (Fig. 3).

## Discussion

In the current study we demonstrated that despite the anticoagulation the RPVO occurs in a large proportion of acute PE patients. Given limited value of the available clinical and laboratory predictors of RPVO, the current study has identified novel potential markers such as plasma fibrin clot permeability and lysability measured ex vivo, both on admission and after 5–7 days. For the first time we found that patients with elevated E-selectin after 3 months since PE, a marker of endothelial damage, are at high risk of RPVO detection on CT at that time. We observed association of baseline early mortality risk assessment, NT-proBNP, and D-dimer with RPVO, but we failed to observe any impact of oxidative stress or inflammatory markers in this regard. The study provides new insights into complex processes underlying RPVO in post-PE patients by highlighting the effect of abnormal fibrin clot networks in the acute phase of PE which cannot be abolished by anticoagulant therapy. Practical implications of the present observations are worth further research given poor identification of the patients with RPVO following low- to moderate-risk PE.

Despite the availability of effective anticoagulants, especially DOAC, RPVO occurs in a substantial proportion of PE patients [25] reaching up to 66% of patients at 3 months and up to 29% a year after PE [26]. The present percentage of 29.1% is consistent with data from recent years. The routine RPVO imaging screening is not recommended in current European guidelines [27, 28] and the decision on repeat imaging is made based on individual clinical presentation. The detection of RPVO indicates an unfavorable prognosis and is associated with increased risk of all-cause death, recurrent VTE, CTEPH, heart failure, and rehospitalization for cardiac causes in the long-term observation [2, 29, 30]. For this reason, blood markers useful in the selection of PE patients at risk of RPVO, especially on admission, are of interest.

It has been demonstrated that plasma fibrin clot properties are altered in PE patients, including reduced K_s_ and prolonged CLT [6]. Such prothrombotic fibrin clot phenotype has been shown to be associated with higher mortality risk and recurrent PE [7, 18, 31]. The present findings relate reduced K_s_ and prolonged CLT measured at baseline and after 5–7 days of anticoagulation with the RPVO occurrence after 3 months with moderate discriminative value. Noteworthy, no similar intergroup differences in fibrin clot properties were observed after 3 months. The RPVO-related differences in K_s_ and CLT cannot be explained by fibrinogen concentrations, the key determinant of fibrin clot measures [32]. However, the fibrinogen molecule is prone to various posttranslational modifications, which involve phosphorylation, hydroxylation, sulfation, oxidation, or nitration and can unfavorably change fibrin clot characteristics [33, 34]. Moreover, since about 500 proteins can be identified within fibrin clots prepared of plasma obtained from VTE patients [35], any changes in their concentrations or activity can affect fibrin clot structure and function. Denser fibrin networks with impaired plasmin-mediated lysis might suggest that obstructed small arteries do not undergo effective recanalization and remained not patent for a few months on imaging studies even if the clinical manifestations like dyspnea are not reported. To our knowledge, observation that K_s_ associates with RPVO is novel, while our finding regarding CLT is in line with the study by Lami et al. [1]. Nevertheless, hypofibrinolysis on admission was not an independent predictor of RPVO at 3 months indicating the stronger impact of compact fibrin networks generated at the same time.

Of particular interest is E-selectin as a marker of RPVO with acceptable accuracy when determined at 3 months of anticoagulation following acute PE. Moreover, a 3-month E-selectin assessed together with baseline K_s_ provides interesting discriminatory alternative for prediction of RPVO. We documented that IL-6 can at least in part drive E-selectin expression and the subsequent increase in its concentrations in circulating blood, which agrees with previous reports [36]. Despite the similar frequency of post-PE syndrome in the RPVO and non-RPVO groups, we also showed that the coexistence of RPVO and post-PE syndrome is associated with the highest E-selectin levels and most unfavorable fibrin clot properties. This supports the hypothesis of a significant role of RPVO in the pathogenesis of post-PE syndrome [3]. Taking into account our results suggesting the involvement of E-selectin in RPVO, the use of E-selectin inhibitor could be an attractive option in PE patients [11]. The completed phase I and II clinical trials with E-selectin inhibitor (GMI-1271) in DVT patients have shown high efficiency without clinically significant differences in coagulation measures in comparison with a low-molecular-weight heparin [37, 38]. However, E-selectin inhibition should be further tested in large randomized clinical trials with evaluation of long-term sequelae of PE.

We have demonstrated that RPVO patients were characterized by higher D-dimer levels at baseline, but not at follow-up. Moreover, higher D-dimer concentrations were an independent predictor of RPVO occurrence. A prognostic value of D-dimer in clinical practice has been shown in previous studies [39]. A meta-analysis by Bruinstroop et al. reported that elevated D-dimer levels measured 1 month after discontinuation of oral anticoagulation predicted VTE recurrence [40]. However, in the previous studies focused on RPVO predictors, the association with D-dimer level has not been convincingly shown [1, 2, 4]. Of note, Kaczyńska et al. demonstrated in 55 patients with first PE on anticoagulation that elevated D-dimer concentration at 6 months, but not on admission, identifies incomplete recanalization of pulmonary artery thromboemboli [41]. Moreover, it has been shown in a subsequent study that a significant decrease in D-dimer level within the first month of anticoagulation was associated with complete pulmonary recanalization [42]. Those findings including ours may indicate that increased blood coagulation activity, reflected by D-dimer levels, is implicated in development of RVPO and is likely modulated by fibrinolytic capacity [41, 42].

Our study has several limitations. First, the sample size was limited though well characterized and representative for normotensive acute PE patients. Second, due to the adopted inclusion and exclusion criteria the results cannot be extrapolated to high-risk PE patients and those with active cancer [43, 44]. Third, some patients did not have all laboratory parameters assessed in the three time points, and the use of anticoagulants was evaluated based on medical records with patient declarations, though suboptimal compliance cannot be ruled out. Moreover, the proposed cut-off values for baseline K_s_ and 3-month E-selectin should be validated in an independent cohort. Finally, a long-term prognostic value of the detected differences in K_s_, CLT, and E-selectin is needed to be evaluated, in particular in the risk of pulmonary hypertension and recurrent PE.

## Conclusions

Despite anticoagulation the RPVO occurs in a significant proportion of low or moderate risk PE patients. We identified novel risk factors of RPVO, namely abnormal fibrin clot characteristics, including formation of compact fibrin networks during the acute PE phase as well as high E-selectin levels after 3 months since the event. Measurement of the parameters, if reliably standardized and validated in an independent cohort, might help select the PE patients who should undergo closer clinical surveillance, due to heightened probability of RPVO and post-PE syndrome.

### Electronic supplementary material

Below is the link to the electronic supplementary material.

Supplementary Material 1
