# Supplementary material for: Reduced fibrin clot permeability on admission and elevated E-selectin at 3 months as novel risk factors of residual pulmonary vascular obstruction in patients with acute pulmonary embolism
Source: J Thromb Thrombolysis. 2023 Nov 6;57(2):248–59. doi: 10.1007/s11239-023-02901-y (PMC10869393; doi:10.1007/s11239-023-02901-y)
Supplement: Supplementary file 1 — Supplementary Material 1 [file 11239_2023_2901_MOESM1_ESM.docx]

Supplementary Table 1. The cut-off values for baseline Ks and 3-month E-selectin in prediction of RPVO with their accuracy.

| Parameter | Cut-off value | Number of patients with: | RPVO, n=23  N (%) | Non-RPVO, n=56  N (%) | Accuracy in prediction of RPVO | % |
| --- | --- | --- | --- | --- | --- | --- |
| Baseline  Ks | 6.55x10^-9^cm^2^ | < cut-off | 21 (26.6) | 9 (11.4) | Sensitivity | 91.3 |
|  |  | ≥ cut-off | 2 (2.5) | 47 (59.5) | Specificity | 83.9 |
| 3-month  E-selectin | 20.25 ng/ml | **>** cut-off | 22 (27.8) | 3 (3.8) | Sensitivity | 95.6 |
|  |  | ≤ cut-off | 1 (1.3) | 53 (67.1) | Specificity | 94.6 |

Abbreviations: RPVO, residual pulmonary vascular obstruction; Ks, plasma fibrin clot permeability.
